# The relationship between dietary inflammatory index and psychosomatic complaints profiles: results from SEPAHAN cross-sectional study

**DOI:** 10.1186/s13030-019-0169-9

**Published:** 2019-11-06

**Authors:** Fahimeh Haghighatdoost, Awat Feizi, Ahmad Esmaillzadeh, Christine Feinle-Bisset, Ammar Hassanzadeh Keshteli, Hamidreza Roohafza, Hamid Afshar, Peyman Adibi

**Affiliations:** 1 Food Security Research Center, Isfahan University of Medical Sciences, Isfahan, Iran; 2 Department of Community Nutrition, School of Nutrition and Food Science, Isfahan University of Medical Sciences, Isfahan, Iran; 3 Psychosomatic Research Center, Isfahan University of Medical Sciences, Isfahan, Iran; 4 Department of Biostatistics and Epidemiology, School of Health, Isfahan University of Medical Sciences, Isfahan, Iran; 5 Integrative Functional Gastroenterology Research Center, Isfahan University of Medical Sciences, Isfahan, Iran; 6 Department of Community Nutrition, School of Nutritional Sciences and Dietetics, Tehran University of Medical Sciences, Tehran, Iran; 7 University of Adelaide, Discipline of Medicine and National Health and Medical Research Council Centre of Research Excellence of Translating Nutritional Science to Good Health, Adelaide, Australia; 8 Department of Medicine, University of Alberta, Edmonton, Canada; 9 Cardiac Rehabilitation Research Center, Cardiovascular Research Institute, Isfahan University of Medical Sciences, Isfahan, Iran

**Keywords:** Dietary inflammatory index (DII), Psychosomatic complaints, Mental health, Psychological disorders

## Abstract

**Background:**

Although the dietary inflammatory index (DII) has been evaluated in relation to psychological disorders risk, the association between DII and psychosomatic complaints is unclear. This study aimed to determine the association between DII, as a proxy measure of the inflammatory potential of the diet, and psychosomatic complaints.

**Methods:**

This cross-sectional study included 2818 people from the general population, aged 19 to 69 years, of Isfahan province in Iran. Dietary intake was assessed using a validated dish-based food-frequency questionnaire. Psychosomatic complaints were assessed using a self-administered validated Persian questionnaire. Twenty-seven nutrients or foods were included in the calculation of DII. Each of them received a score based on their inflammatory ability, thus, a higher DII score indicated a more pro-inflammatory diet. The risk of being in the top median of a psychosomatic complaints profile across the tertiles of DII was assessed using logistic regression.

**Results:**

Four psychosomatic complaints profiles (psychological, gastrointestinal, neuro-skeletal and pharyngeal- respiratory) were identified. After controlling for potential confounders, individuals in the top tertile of DII had higher odds for experiencing high somatic complaints scores for the psychological (odds ratio (OR) = 1.44, 95% confidence interval (CI): 1.10, 1.89; P trend = 0.009), gastrointestinal (OR = 1.22, 95% CI: 0.99, 1.75; *P* = 0.058) or neuro-skeletal (OR = 1.56, 95% CI: 1.10, 2.22; *P* = 0.013) profile. The association for pharyngeal-respiratory complaints did not remain significant after adjustment for stressful life events, medical condition, and anti-psychotropic medicine use.

**Conclusion:**

The significant positive link between DII and the various psychosomatic complaints profiles suggests that a diet with anti-inflammatory potential might be favourably related to psychosomatic complaints. Further studies, particularly clinical trials and longitudinal studies, are warranted to investigate the inflammatory potential of diet in relation to psychosomatic complaints.

## Introduction

According to the Japanese Society of Psychosomatic Medicine, psychosomatic illness refers to any organic or functional damage as a result of psychological disorders [1]. Psychosomatic illness has a substantial effect on quality of life [2, 3]. In view of the growing prevalence of psychological disorders [4], along with their large impacts on medical history of diseases, it is necessary to identify new approaches to prevent psychological disorders and their medical complications, or delay their progression.

Peripheral and central inflammatory pathways have been positively linked to mental disorders [5], as has functional damage in somatization [6]. Recent evidence suggests that nutritional interventions can be considered as a mainstream approach in psychiatric practice [7, 8]. Recently, results of a meta-analysis have shown that a ‘healthy dietary pattern’, characterized by whole grains, fruits and vegetables, is associated with a lower risk of depression and anxiety, whereas an ‘unhealthy dietary pattern’, which consisted of red or processed meats, refined grains, and high-sugar or high-fat foods, is associated with an increased risk of mental disorders [9].

In addition, various bioactive components in the diet may exert pro- or anti-inflammatory effects [10]. Therefore, pro-inflammatory features of an unhealthy dietary pattern may underpin chronic inflammation [11–13], psychological disorders [14], and probably psychosomatic complaints. Several investigations have evaluated the relation between the dietary inflammatory index (DII), reflective of the potential pro-inflammatory properties of a diet, with psychological disorders, with most indicating a direct link between DII and the risk of psychological disorders [15–22].

In a recent analysis based on data from the SEPAHAN project, and consistent with earlier publications [15–22], we found that higher DII scores were associated with an increased risk of having higher psychological disorders profile scores (including depression, anxiety and psychological distress) [23]. Moreover, in another study in this population, we found that a healthy dietary pattern was inversely associated with the risk of all identified psychosomatic, including psychological, gastrointestinal, neuro-skeletal and pharyngeal-respiratory somatic, complaints, whilst greater adherence to the Western (unhealthy) dietary pattern was associated with a greater risk of psychological somatic complaints [24].

Although several studies have assessed the relation between DII and psychological disorders, we are not aware of any study investigating the relation between DII and psychosomatic complaints profiles. Due to the considerable impact of psychological status on organic and functional disorders, as well as the effect of diet and inflammation on mood status, we hypothesized that a pro-inflammatory diet may influence somatic complaints by affecting mood status. Therefore, the current study was conducted to examine the association between the dietary inflammatory index and psychosomatic complaints profiles in a large sample of Iranian adults.

## Methods and materials

### Study population

Data were included from the Study on the Epidemiology of Psychological, Alimentary Health and Nutrition (SEPAHAN) project. SEPAHAN is a cross-sectional study of lifestyle and psychological risk factors of common gastrointestinal disorders. Within the SEPAHAN project, non-academic members of the staff of Isfahan University of Medical Sciences (IUMS), Isfahan, Iran, working on the university campus and in 50 hospitals and health centers affiliated with IUMS, were recruited in two separate phases. The first phase commenced in April 2010, and 10,087 questionnaires were distributed among participants at this phase. Participants were asked to return completed questionnaires within the next 3 weeks. The response rate in this phase was 86.16%; i.e. 8691 completed questionnaires were returned. The second phase was run in mid-May 2010, and information regarding common gastrointestinal symptoms and their psychological profile was collected in this phase. 6516 completed questionnaires were returned within 2 weeks (response rate = 64.6%.) Finally, 4763 questionnaires in phase 2 could be matched with their corresponding questionnaires in phase 1. In the current analysis, 2818 persons, aged from 19 to 69 years, whose energy intake was in the range of 800 to 4200 kcal/d [25] and who had complete information regarding both dietary factors and somatoform symptoms, were enrolled. All participants provided written informed consent, and the study protocol was approved by the Regional Bioethics Committee of IUMS (#189069, #189082, and #189086). Detailed information about the methodology used in the SEPAHAN project has been described elsewhere [26]. The current secondary study project number is 297179 with ethical approval code: IR.MUI.MED.REC.1398.025.

### Dietary intake assessment

Dietary intake over the previous year was assessed using a validated, self-administered 106-item dish-based food-frequency questionnaire (DFQ). The DFQ was designed according to the Willet-format food-frequency questionnaire. Details regarding its design, validity, and reliability have been described elsewhere [27]. In brief, all food items were categorized into five main categories, including mixed dishes (cooked or canned, *n* = 29); grain-based foods and potatoes (*n* = 10); dairy products (milk and dairy products, incl. Butter and cream, *n* = 9); fruit and vegetables (*n* = 22); and miscellaneous food items and beverages (including sweets, fast foods, nuts, desserts, and beverages, *n* = 36). To determine the amount of food consumed, the portion sizes most common to all people were provided for each food item. The frequency of consumption was rated at nine levels, from “never or less than once a month” to “12 or more times per day”. This structure differed according to the popularity of food items. For example, for foods that are consumed less frequently, high frequency items were omitted, but kept for foods that are consumed regularly. Food items were converted to grams/d using household measures [28]. Daily energy and nutrient intakes of each participant were estimated based on the USDA food composition database [29].

### Calculation of the dietary inflammatory index

The DII was created based on the method suggested by Shivappa et al. [10]. Accordingly, all food/nutrient parameters included in the DII were adjusted for energy intake using the residual method, as suggested by Willett and Stampfer [30]. Next, each food parameter was linked to its regionally representative database that provides an estimate of global mean intake for each parameter, along with its reported standard deviation in the DII definition [10]. Then, the standard global mean, derived from eleven different populations around the world, was subtracted from the actual dietary intake in the study population and divided by its standard deviation to calculate z scores. All z scores were converted to percentiles and then doubled and “1” was subtracted to centralize percentiles. Finally, these values were multiplied by their corresponding overall inflammatory effect score and summed to create the overall DII. Some food components were not included in the calculation of the DII in the present study, because they are not used in the Iranian diet (e.g. ethanol) or data were not available (e.g. some spices). Thus, in the current study, the DII was calculated based on 27 nutrients (energy, carbohydrate, protein, total fat, cholesterol, monounsaturated fatty acids, polyunsaturated fatty acids, saturated fat, n-3 fatty acids, n-6 fatty acids, trans fat, fiber, thiamin, riboflavin, niacin, vitamin B6, folic acid, vitamin B12, vitamin C, vitamin A, vitamin D, vitamin E, β-carotene, iron, magnesium, selenium, and zinc), onions, tea, and caffeine, which were derived from the DFQ. Higher DII scores indicated a greater pro-inflammatory potential of the diet.

### Assessment of somatization

Somatization was defined according to the standard 47-item questionnaire developed by Lacourt et al. [31]. However, since 16 questions of this instrument and the severity of symptoms were not assessed in the SEPAHAN project, functional somatization symptoms in the current study were defined based on 31 questions and the frequency of symptoms rather than their severity. These 31 questions were common between SEPAHAN’s questionnaires and Lacourt. The patient health questionnaire (PHQ) was also used as a standard instrument to evaluate somatic complaints [31–33]. Participants were asked about the frequency of experiencing each symptom during the past 3 months. Each question was answered using a four-point Likert scale (1 = never or rarely, 2 = sometimes, 3 = often, 4 = always), except for two questions regarding pain in teeth or jaw and dry mouth, which used a three-point Likert scale (1 = never, 2 = low and 3 = high). The “medically explained” and “unexplained” symptoms [34] are the relevant features of the somatization concept [35, 36]. However, only gastrointestinal symptoms were evaluated using adequate clinical and technical approaches to provide a medical explanation, whilst other somatic symptoms are only indicative of somatization. More detail, including on the validity and reliability of the instrument to assess somatoform symptoms profile, has been provided in our earlier publications [37, 38]. Briefly, a mini survey of 100 randomly selected participants illustrated a strong reliability, with a Cronbach’s alpha score of 0.903 [37, 38].

### Assessment of other covariates

Data on age (y), sex (male/female), marital status (married/single), education (≤12, 12–16, > 16 y), weight (Kg) and height (m), and lifestyle data, such as smoking habits (current or ex-smoker/non-smoker), was self-reported and physical activity (inactive or moderately inactive/moderately active or active) was assessed using pretested general practice activity questionnaire (GPAQ) [39]. Body mass index (BMI) was calculated by dividing weight (kg) by height squared (m2). Participants also provided information about their medical history of diseases, including any history of hyperlipidemia, hypertension, diabetes mellitus, asthma, stroke, myocardial infarction, gastrointestinal bleeding, gallstone, cancer, Crohn’s disease and ulcerative colitis, as well as the use of psychotropic and gastrointestinal medications (since the SEPAHAN project aimed to examine the association between lifestyle factors with mental and gastrointestinal disorders). Stressful life events over the past 6 months were assessed using a validated stressful life events (SLE) questionnaire [40]. The total stress score ranged from 0 to 83, with higher scores indicating more severe stressful life events. This score was treated as a continuous covariate in the statistical analysis, as described [40].

### Statistical analysis

In total, 31 items (symptoms) were considered in the determination of the psychosomatic complaints profiles using exploratory factor analysis and a principal component extraction approach. A simple and interpretable structure was obtained by the Varimax orthogonal transformation method. The interpretability of factors, eigenvalues> 1 and the Scree test determined which factors should be retained. The correlations between each item and derived psychosomatic complaints profiles were determined using factor loadings with a cut-off value of 0.2. Identified factors were labeled based on the items highly loaded in each factor [37, 38]. Considerable decrement in eigenvalues was observed after the fourth factor and remained approximately similar for other identified factors after the fifth one. To calculate each participant’s factor score, all of the items used in the identification of patterns were weighted by their factor loadings and then summed. Thus, each participant received a factor score for each identified psychosomatic complaint profile, which was then categorized into high (scores more than median) or low (scores lower than median) based on the median value. Accordingly, participants were categorized as suffering from high levels of each specific psychosomatic complaints profile when they were placed in the high median group. Data for continuous and categorical variables are reported as mean and standard error (SE) or percentages across tertiles of DII, respectively. Differences among groups were examined using analysis of variance (ANOVA) for continuous variables and chi-square test for categorical variables. Food groups and nutrients were adjusted for age (y), sex, and total energy intake (kcal/d) and compared between tertiles of the dietary inflammatory index by analysis of covariance (ANCOVA). Total energy intake was adjusted for age and sex. Correlation coefficients between DII’s components and identified somatic complaints profiles’ scores were measured using non-parametric Spearman rank correlation coefficient. Odds ratios (ORs) and 95% confidence intervals (CIs) for being in the high median of psychosomatic complaints profiles scores were estimated through binary logistic regression in crude and multivariable-adjusted models. We also controlled for some confounding covariates which could potentially affect either the exposure (dietary intakes) or outcomes (psychosomatic complaints). In the first adjusted model (model 1), the confounding effects of age, sex, and energy (kcal/d) were controlled. Although nutrient residuals were used in the calculation of the DII, we also controlled for total calorie intake, because of its potential to affect psychosomatic complaints [41]. In model 2, additional adjustment was made for marital status, education, smoking, and physical activity. Further adjustment for BMI was made in model 3. Finally, the effects of stressful life event, anti-psychotropic medicine (yes/no) and medical history of chronic disease were additionally adjusted in model 4. *P* for linear trend was determined through entering tertiles of DII as a continuous variable in the logistic regression model. Means of the psychosomatic complaints profile were compared between men and women using independent samples t-test in the crude model and using ANCOVA in the adjusted model in the framework of general linear model (GLM). When there was a significant difference between men and women, stratified analysis by sex was performed. Stratified analysis by sex, applying the above-mentioned adjusted models, was run to examine potential modifying effect of sex in relation to the association of DII and psychosomatic complaints profiles. Statistical analyses were performed using the Statistical Package for Social Sciences (SPSS, Inc., Chicago IL, USA; version 16). *P* < 0.05 was considered significant in all analyses.

## Results

Table 1 shows age-, sex- and energy-adjusted dietary intakes of the study population according to tertiles of DII. Compared with those in the lowest tertile, those in the highest tertile of DII had greater intakes of energy, fat, caffeine, hydrogenated vegetable oils, and refined grains, but lower intakes of carbohydrate, protein, fiber, folate, pyridoxine, magnesium, fruit, vegetables, nuts, legumes and soy, red and white meats and whole grains.

**Table 1:** Dietary intakes of the participants across tertiles of the dietary inflammatory index^1^

Variables	Tertiles of the dietary inflammatory index			*P* value^2^
	1 (−5.55, −2.60)	2 (−2.61, − 1.52)	3 (− 1.53, 4.61)	
n	939	940	939	
Energy (kcal/d)	2040.02 ± 25.94	2273.71 ± 25.67	2844.32 ± 26.05	< 0.0001
Carbohydrate (% of total daily energy)	50.64 ± 0.28	48.41 ± 0.27	48.42 ± 0.29	< 0.0001
Fat (% of total daily energy)	36.30 ± 0.23	38.07 ± 0.22	38.18 ± 0.24	< 0.0001
Protein (% of total daily energy)	15.29 ± 0.08	14.93 ± 0.08	14.22 ± 0.08	< 0.0001
Fiber (g/d)	27.12 ± 0.16	22.96 ± 0.16	17.67 ± 0.17	< 0.0001
Caffeine (mg/d)	88.37 ± 3.27	96.49 ± 3.14	112.97 ± 3.37	< 0.0001
Total folate intake (μg/d)	604.36 ± 4.28	569.21 ± 4.11	549.68 ± 4.41	< 0.0001
Vitamin B6 (mg/d)	2.24 ± 0.01	2.01 ± 0.01	1.69 ± 0.01	< 0.0001
Vitamin B12 (μg/d)	2.99 ± 0.04	3.00 ± 0.04	2.90 ± 0.04	0.101
Mg (mg/d)	366.34 ± 1.68	333.74 ± 1.61	284.99 ± 1.73	< 0.0001
Omega-3 fatty acids (g/d)	2.28 ± 0.04	2.25 ± 0.04	2.23 ± 0.04	0.648
Food groups				
Fruit (g/d)	452.98 ± 7.53	311.71 ± 7.23	196.13 ± 7.76	< 0.0001
Vegetables (g/d)	322.29 ± 3.74	235.29 ± 3.59	161.13 ± 3.85	< 0.0001
Nuts, legumes and soy (g/d)	65.72 ± 1.24	58.79 ± 1.19	43.07 ± 1.27	< 0.0001
White meat (g/d)	66.27 ± 1.55	64.54 ± 1.48	60.46 ± 1.59	0.039
Red meat (g/d)	83.02 ± 1.40	82.09 ± 1.35	69.79 ± 1.44	< 0.0001
Hydrogenated vegetable oil (g/d)	9.67 ± 0.38	10.52 ± 0.37	11.36 ± 0.39	0.014
Refined grains (g/d)	338.03 ± 5.67	375.38 ± 5.44	460.88 ± 5.84	< 0.0001
Whole grains (g/d)	57.00 ± 2.69	51.73 ± 2.58	23.19 ± 2.77	< 0.0001

^1^Values are Mean ± SE. Nutrients were adjusted for age, sex and total energy intake (kcal). Energy intake was adjusted for age and sex. Tertile 1: low pro-inflammatory properties, tertile 2: medium pro-inflammatory properties and tertile 3: high pro-inflammatory properties

^2^From analysis of covariance (ANCOVA), with energy considered as the absolute amount per day

Table 2 shows the individual somatic complaints factor loading matrix for each of the four psychosomatic complaints. Each psychosomatic complaint profile was labeled based on individual somatic complaints which were highly loaded. For example, disorders related to feelings, thoughts, and sleep were captured in the ′psychological′ somatic complaints profile, complaints related to the gastrointestinal tract, including nausea, diarrhea, and bloating, in the ′gastrointestinal′ profile, back, neck, and joint pain in the ′neuro-skeletal′ profile, and disorders relating to the pharyngeal and respiratory systems, such as shortness of breath, hoarseness and wheezing, in the ′pharyngeal-respiratory′ profile. The ′psychological′ somatic complaints explained 12.7% of the total variance. Corresponding values for ′gastrointestinal′, ′neuro-skeletal′, and ′pharyngeal-respiratory′ were 11.7, 11.6, and 8.9%, respectively [37, 38]. The correlation coefficients of the identified psychosomatic complaints profiles with DII and its individual components are shown in Additional file 1: Table S1. In most cases, correlation coefficients were greater for DII compared with correlations for its components. However, correlations between all four psychosomatic complaints and vitamin C were constantly greater than those for DII; therefore, the risk of psychosomatic complaints were assessed across the tertiles of vitamin C intake (Additional file 1: Table S2). In spite of an inverse association between vitamin C intake and all four somatic complaints in the crude model, the associations remained significant only for pharyngeal-respiratory somatic complaints and showed a trend towards lower risk for psychological and gastrointestinal somatic complaints in the fully-adjusted model.

**Table 2:** Factor loadings for the four extracted somatic complaints profiles from 31 somatic complaints

Somatic complaints	Factor loadings ^a^			
	Psychological	Gastrointestinal	Neuro-skeletal	Pharyngeal-respiratory
Sleep disorder	**0.420**	0.148	0.325	0.154
Pounding heart	**0.577**	0.129	0.300	0.236
Feeling low on energy	**0.565**	0.163	0.416	0.014
Feeling like ′butterflies′ in the stomach	**0.869**	0.242	0.110	0.167
Difficulty concentrating	**0.869**	0.242	0.110	0.167
Disturbing thoughts	**0.664**	0.144	0.261	0.036
Dry mouth	0.103	**0.248**	0.236	0.220
Chest pain	0.339	**0.454**	0.087	0.233
Feeling of fullness	0.259	**0.662**	0.139	0.132
Nausea	0.283	**0.509**	0.047	0.262
Gastroesophageal reflux	0.153	**0.544**	0.012	0.318
Pain or discomfort in the abdomen	0.239	**0.678**	0.206	0.139
Constipation	0.070	**0.512**	0.255	−0.037
Diarrhea	−0.061	**0.374**	0.235	0.124
Bloating or swelling of the abdomen	0.210	**0.644**	0.288	0.022
Anal pain	0.122	**0.473**	0.239	0.183
Headache	0.246	0.239	**0.547**	0.134
Back pain	0.139	0.168	**0.661**	0.148
Pain in joints	0.230	0.170	**0.612**	0.160
Eyesore	0.207	0.075	**0.461**	0.301
Severe fatigue	0.361	0.185	**0.646**	0.081
Dizziness and confusion	0.343	0.245	**0.513**	0.254
Chills and extreme cold	0.158	0.175	**0.435**	0.346
Hot flashes	0.274	0.279	**0.350**	0.146
Menstrual disorder	−0.038	0.323	**0.389**	−0.021
Neck pain	0.105	0.154	0.322	**0.570**
Globus sensation	0.165	0.372	0.015	**0.510**
Having trouble swallowing	0.090	0.240	−0.021	**0.596**
Shortness of breath	0.391	0.124	0.311	**0.492**
Hoarseness	0.015	0.092	0.225	**0.612**
Wheezing (asthma)	0.104	0.000	0.124	**0.567**
Variance explained (%)	12.70	11.73	11.55	8.85
Cumulative variance	12.70	24.43	35.99	44.84

^a^The highest factor loadings have been highlighted, and the interpretation of factors was done based on them

Participant characteristics across the tertiles of DII are presented in Table 3. Participants with the highest DII scores, reflecting a more pro-inflammatory diet, were more likely to be younger, male, and current smokers, whilst those in the lowest tertile were more likely to be physically active and overweight or obese. Marital status and education levels did not differ across the tertiles of DII.

**Table 3:** General characteristics of the study participants across tertiles of the dietary inflammatory index^1^

Variables	Tertiles of the dietary inflammatory index			*P* value^2^
	1 (−5.55, −2.60)	2 (−2.61, −1.52)	3 (−1.53, 4.61)	
n	939	940	939	
Age (years)	37.6 ± 0.3	36.0 ± 0.3	35.4 ± 0.3	< 0.0001
BMI (kg/m^2^)	25.5 ± 0.2	24.9 ± 0.1	24.7 ± 0.1	< 0.0001
Male (%)	34.0	40.7	48.1	< 0.0001
Married (%)	83.3	81.2	78.5	0.121
Educational level (%)				0.888
≤12 yrs	11.6	10.0	10.3	
12–16 yrs	80.3	82.1	81.2	
> 16 yrs	8.1	7.8	8.4	
Physically active (%)	52.4	44.9	41.2	< 0.0001
Overweight or obese^3^ (%)	51.3	45.2	43.8	0.003
Current smokers (%)	13.5	12.4	14.1	0.020

*BMI* body mass index

^1^Values are Mean ± SE unless otherwise indicated. Tertile 1: low pro-inflammatory properties, tertile 2: medium pro-inflammatory properties and tertile 3: high pro-inflammatory properties

^2^Resuls from one-way ANOVA and χ^2^ test for continuous and categorical variables, respectively

^3^Overweight was defined as BMI ≥25 and ≤ 29.99 kg/m^2^ and obese was defined as BMI ≥ 30 kg/m^2^

Logistic regression analysis (Table 4) indicated that, in the crude model, individuals in the top tertile of DII had a greater risk of having higher scores for psychological (OR = 1.40, 95% CI: 1.16, 1.69; *P* < 0.0001), neuro-skeletal (OR = 1.35, 95% CI: 1.06, 1.73; *P* = 0.019), and pharyngeal-respiratory (OR = 1.22, 95% CI: 1.01, 1.48; *P* = 0.035) somatic complaints compared with those in the first tertile, while, despite a trend towards a higher risk of having greater scores for gastrointestinal somatic complaints with a more pro-inflammatory diet, the association did not reach significance (OR = 1.22, 95% CI: 0.99, 1.49; *P* = 0.058). Adjustment for age, sex, and energy intake, lifestyle factors and BMI strengthened the risk of having higher scores of psychological (OR = 1.65, 95% CI: 1.29, 2.12; *P* < 0.0001), gastrointestinal (OR = 1.49, 95% CI: 1.14, 1.94; *P* = 0.003), neuro-skeletal (OR = 1.69, 95% CI: 1.22, 2.34; *P* = 0.002), and pharyngeal-respiratory (OR = 1.37, 95% CI: 1.07, 1.74; *P* = 0.011) somatic complaints for those in the highest tertile of DII compared with those in the lowest tertile. Moreover, after adjustment for stressful life events, medical history, and anti-psychotropic medicines in the fully-adjusted model, the observed associations between DII and all somatic complaints profile remained significant except for pharyngeal-respiratory (OR = 1.23, 95% CI: 0.96, 1.58; *P* = 0.102).

**Table 4:** Crude and multivariable-adjusted odds ratio and 95% confidence interval for the psychosomatic complaints profiles across tertiles of the dietary inflammatory index

	Tertiles of the dietary inflammatory index			P trend^1^
	1 (−5.55, − 2.60)	2 (−2.61, − 1.52)	3 (−1.53, 4.61)	
n	939	940	939	
Psychological somatic complaints profile				
Crude	1 (Reference)	1.04 (0.86, 1.25)	1.40 (1.16, 1.69)	< 0.0001
Model 1	1 (Reference)	1.16 (0.95, 1.43)	1.69 (1.34, 2.12)	< 0.0001
Model 2	1 (Reference)	1.10 (0.88, 1.37)	1.65 (1.29, 2.10)	< 0.0001
Model 3	1 (Reference)	1.09 (0.87, 1.37)	1.65 (1.29, 2.12)	< 0.0001
Model 4	1 (Reference)	1.05 (0.82, 1.34)	1.44 (1.10, 1.89)	0.009
Gastrointestinal somatic complaints profile				
Crude	1 (Reference)	1.12 (0.91, 1.37)	1.22 (0.99, 1.49)	0.058
Model 1	1 (Reference)	1.24 (0.99, 1.54)	1.50 (1.18, 1.92)	0.001
Model 2	1 (Reference)	1.23 (0.97, 1.55)	1.45 (1.12, 1.88)	0.005
Model 3	1 (Reference)	1.24 (0.98, 1.58)	1.49 (1.14, 1.94)	0.003
Model 4	1 (Reference)	1.21 (0.94, 1.56)	1.32 (1.00, 1.75)	0.048
Neuro-skeletal somatic complaints profile				
Crude	1 (Reference)	1.00 (0.79, 1.27)	1.35 (1.06, 1.73)	0.019
Model 1	1 (Reference)	1.08 (0.84, 1.39)	1.59 (1.18, 2.13)	0.003
Model 2	1 (Reference)	1.19 (0.90, 1.57)	1.66 (1.21, 2.28)	0.002
Model 3	1 (Reference)	1.20 (0.90, 1.59)	1.69 (1.22, 2.34)	0.002
Model 4	1 (Reference)	1.23 (0.90, 1.67)	1.56 (1.10, 2.22)	0.013
Pharyngeal -respiratory somatic complaints profile				
Crude	1 (Reference)	1.14 (0.94, 1.38)	1.22 (1.01, 1.48)	0.035
Model 1	1 (Reference)	1.14 (0.93, 1.39)	1.29 (1.04, 1.62)	0.023
Model 2	1 (Reference)	1.18 (0.95, 1.46)	1.32 (1.04, 1.68)	0.022
Model 3	1 (Reference)	1.17 (0.94, 1.46)	1.37 (1.07, 1.74)	0.011
Model 4	1 (Reference)	1.14 (0.91, 1.43)	1.23 (0.96, 1.58)	0.102

^1^From Mantel-Haenszel extension chi-square test. Tertile 1: low pro-inflammatory properties, tertile 2: medium pro-inflammatory properties, and tertile 3: high pro-inflammatory properties

Model 1: adjustment was made for age, sex, and energy (kcal/d). Model 2: additional adjustment was made for marital status, education, smoking, and physical activity. Model 3: adjusted for body mass index (BMI). Model 4: anti-psychotropic medicines, medical history of diseases, and stressful life events were additionally adjusted

Means of psychosomatic complaints profiles as well as mean value of DII illustrated significant differences between men and women (Additional file 1: Table S3); indicating sex and DII affect psychosomatic complaints profiles interactively and, therefore, the analyses were separately performed for men and women. Stratified logistic regression analysis demonstrated a sex-specific association between DII and psychosomatic complaints profiles (Table 5). While in the crude model, a greater risk of having higher scores of psychological somatic complaints was observed among men and women in the highest compared with the lowest tertile of DII, these associations tended to be stronger in the adjusted models for men. In contrast, controlling for anti-psychotropic medicines, medical history, and stressful life events was not associated with a significant relation between DII and the psychological somatic complaints of men, whilst it remained significant in all adjusted models for women. The risk of having higher gastrointestinal somatic complaints scores tended to be higher in the highest DII tertile for both men and women, but the significance disappeared in the fully adjusted model for women. In addition, men in the highest tertile DII had a 70% greater risk of having higher scores of pharyngeal-respiratory somatic complaints in the crude model, and the association remained significant even after adjustment for demographic and lifestyle variables, energy, BMI, stressful life event, anti-psychotropic medicine, and medical history (OR = 1.57, 95% CI: 0.03, 2.40; *P* = 0.033), whereas no significant association was observed for women. In contrast, a higher DII was significantly associated with increased the risk of higher scores of neuro-skeletal somatic complaints both in crude and adjusted models for women, but not for men.

**Table 5:** Crude and multivariable-adjusted odds ratio and 95% confidence interval for the psychosomatic complaints profiles across tertiles of the dietary inflammatory index, stratified by sex

	Tertiles of dietary inflammatory index			P trend^1^
	1	2	3	
Male (n)	319	383	452	
DII range	−5.55, −2.62	−2.62, −1.52	−1.52, 2.62	
Psychological somatic complaints profile				
Crude	1 (Reference)	1.22 (0.88, 1.68)	1.46 (1.07, 1.98)	0.017
Model 1	1 (Reference)	1.29 (0.90, 1.84)	1.50 (1.03, 2.18)	0.034
Model 2	1 (Reference)	1.18 (0.80, 1.73)	1.41 (0.94, 2.11)	0.094
Model 3	1 (Reference)	1.15 (0.78, 1.70)	1.43 (0.95, 2.15)	0.082
Model 4	1 (Reference)	1.03 (0.67, 1.59)	1.16 (0.74, 1.81)	0.514
Gastrointestinal somatic complaints profile				
Crude	1 (Reference)	1.17 (0.83, 1.65)	1.37 (0.99, 1.90)	0.059
Model 1	1 (Reference)	1.32 (0.91, 1.93)	1.58 (1.07, 2.35)	0.023
Model 2	1 (Reference)	1.34 (0.89, 2.01)	1.61 (1.05, 2.47)	0.030
Model 3	1 (Reference)	1.37 (0.91, 2.07)	1.70 (1.10, 2.63)	0.017
Model 4	1 (Reference)	1.36 (0.88, 2.12)	1.51 (0.94, 2.41)	0.089
Neuro-skeletal somatic complaints profile				
Crude	1 (Reference)	1.04 (0.37, 2.91)	1.42 (0.52, 3.88)	0.481
Model 1	1 (Reference)	1.52 (0.37, 6.15)	0.72 (0.11, 4.49)	0.919
Model 2	1 (Reference)	1.41 (0.29, 6.99)	0.83 (0.10, 6.91)	0.959
Model 3	1 (Reference)	1.35 (0.24, 7.73)	0.84 (0.09, 7.74)	0.953
Model 4	1 (Reference)	7.03 (0.45, 110.1)	0.48 (0.03, 8.56)	0.902
Pharyngeal -respiratory somatic complaints profile				
Crude	1 (Reference)	1.29 (0.94, 1.77)	1.70 (1.25, 2.30)	0.001
Model 1	1 (Reference)	1.28 (0.90, 1.83)	1.68 (1.17, 2.43)	0.005
Model 2	1 (Reference)	1.28 (0.87, 1.88)	1.69 (1.13, 2.51)	0.009
Model 3	1 (Reference)	1.26 (0.86, 1.86)	1.76 (1.18, 2.64)	0.005
Model 4	1 (Reference)	1.19 (0.80, 1.79)	1.57 (1.03, 2.40)	0.033
Female (n)	620	557	487	
DII range	−5.17, −2.61	−2.61, −1.52	−1.52, 4.61	
Psychological somatic complaints profile				
Crude	1 (Reference)	1.02 (0.81, 1.30)	1.73 (1.34, 2.23)	< 0.0001
Model 1	1 (Reference)	1.09 (0.85, 1.40)	1.87 (1.40, 2.51)	< 0.0001
Model 2	1 (Reference)	1.05 (0.80, 1.38)	1.82 (1.33, 2.49)	< 0.0001
Model 3	1 (Reference)	1.05 (0.80, 1.39)	1.79 (1.30, 2.46)	0.001
Model 4	1 (Reference)	1.04 (0.77, 1.40)	1.65 (1.16, 2.34)	0.008
Gastrointestinal somatic complaints profile				
Crude	1 (Reference)	1.14 (0.88, 1.47)	1.27 (0.97, 1.66)	0.076
Model 1	1 (Reference)	1.18 (0.90, 1.56)	1.47 (1.08, 2.02)	0.016
Model 2	1 (Reference)	1.16 (0.87, 1.56)	1.37 (0.98, 1.90)	0.065
Model 3	1 (Reference)	1.17 (0.87, 1.57)	1.38 (0.98, 1.93)	0.063
Model 4	1 (Reference)	1.14 (0.83, 1.56)	1.26 (0.88, 1.80)	0.205
Neuro-skeletal complaints profile				
Crude	1 (Reference)	1.01 (0.79, 1.30)	1.39 (1.08, 1.79)	0.015
Model 1	1 (Reference)	1.07 (0.82, 1.38)	1.61 (1.19, 2.17)	0.003
Model 2	1 (Reference)	1.18 (0.89, 1.57)	1.66 (1.20, 2.30)	0.002
Model 3	1 (Reference)	1.20 (0.90, 1.59)	1.69 (1.21, 2.36)	0.002
Model 4	1 (Reference)	1.21 (0.89, 1.65)	1.58 (1.10, 2.25)	0.013
Pharyngeal -respiratory somatic complaints profile				
Crude	1 (Reference)	1.12 (0.88, 1.41)	1.05 (0.82, 1.34)	0.665
Model 1	1 (Reference)	1.08 (0.84, 1.38)	1.10 (0.83, 1.46)	0.492
Model 2	1 (Reference)	1.14 (0.88, 1.49)	1.14 (0.84, 1.54)	0.361
Model 3	1 (Reference)	1.15 (0.87, 1.50)	1.18 (0.86, 1.60)	0.278
Model 4	1 (Reference)	1.14 (0.87, 1.51)	1.09 (0.79, 1.49)	0.555

^1^From Mantel-Haenszel extension chi-square test. Tertile 1: low pro-inflammatory properties, tertile 2: medium pro-inflammatory properties and tertile 3: high pro-inflammatory properties

Model 1: adjustment was made for age and energy (kcal/d). Model 2: additional adjustment was made for marital status, education, smoking, and physical activity. Model 3: adjusted for body mass index (BMI). Model 4: anti-psychotropic medicines, medical history, and stressful life events were additionally adjusted

## Discussion

To the best of our knowledge, this is the first study examining the association between the dietary inflammatory index and psychosomatic complaints in an adult population. This study suggests a direct link between a pro-inflammatory diet and various psychosomatic complaints profiles, including psychological, gastrointestinal, neuro-skeletal, and pharyngeal-respiratory somatic complaints, in crude and various adjusted models.

Psychosomatic symptoms are attributed to intracellular inflammation, oxidative stress damage, and gut-derived inflammation [6]. Higher levels of nuclear factor kappa beta (NF-κβ) is correlated with psychosomatic symptoms, such as muscular tension, fatigue, pain, sadness, and irritability [6]. Furthermore, increased production of NFκβ, a characteristic of psychological disorders [42–44], stimulates the inflammation pathways through transcriptional activation of inflammatory markers, such as interleukin-6 (IL-6), interleukin-1β (IL-1β), and tumor necrosis factor-α (TNF-α) [6]. Over the last few decades, robust evidence has been accumulated to indicate that elevated inflammatory mediators are a consistent feature of psychiatric diseases [45], and a meta-analysis has suggested a bidirectional link between systematic inflammation and mental disorders [46]. Therefore, developing approaches that target inflammatory pathways are warranted to reduce the incidence of mental disorders and psychosomatic complaints.

To date, many studies have examined the effects of diet composition on serum levels of inflammatory markers [47]. For example, a high glycemic index diet increases NF-κβ activation [48] and stimulates the inflammatory signaling cascade, whilst whole grains [49] and a vegetarian diet [50] may have anti-inflammatory effects. In addition, increased total flavonoid consumption is inversely correlated with serum levels of CRP [51], which have been attributed to an inhibitory effect on the release of cytokines or down-regulation of pro-inflammatory transcriptional factors [52]. Taken together, a number of dietary components have been related to pro- or anti-inflammatory pathways and the circulating concentrations of pro- or anti-inflammatory mediators. Despite this, it has been suggested that taking into account diet as a whole, rather than its individual components, may be important, because of the role of potential interactions between, and/or synergistic effects of food components.

In this regard, the DII has been developed as an index reflecting the inflammatory potential of the whole diet. Correlations between the DII with circulating levels of inflammatory mediators have been found in some [11–13], but not all studies [13, 20]. However, most studies assessing the relation between DII and mental disorders have reported a positive link. In several observational studies, including our own research in this study population, higher DII scores were associated with increased risk of depression or anxiety, or lower likelihood of well-being [15–23]. Due to the close relation between mental disorders and psychosomatic symptoms, an association between DII and psychosomatic complaints is conceivable, and it is expected that amelioration of psychological disorders would improve somatic complaints. However, it is not clear which inflammatory mediators, and to which extent, contribute to developing mental disorders or their somatic symptoms. In the Whitehall II study, despite a significant link between DII and inflammatory biomarkers at baseline, the recurrence of depressive symptoms was not related to the serum concentrations of IL-6 and CRP after a 5-year follow-up [16]. These findings may suggest that diet-related inflammatory effects are mediated via mediators other than IL-6 and CRP. Moreover, since CRP and IL-6 levels are affected by many modifying factors, such as age, adiposity, physical fitness, and activity, they are unlikely to be specific indicators for systematic inflammation per se [53]. Thus, further research investigating the link between DII and inflammatory mediators is required.

Furthermore, in studies investigating DII, the inflammatory potential of diet has consistently been associated with a less healthy nutritional profile [15, 23]. In addition, like a diet with lower potential for inflammation, healthy dietary patterns such as the Mediterranean eating style, healthy eating index, and other healthy patterns identified by posteriori method have been associated with lower serum levels of inflammatory mediators [54, 55]. Hence, in agreement with our previous work [24], the results of the present study confirm lower risk of different psychosomatic complaints profiles among individuals with a healthier dietary pattern, and conversely higher risk of psychosomatic complaints profiles among individuals with a Western dietary pattern, which have been assumed to have anti-inflammatory and pro-inflammatory properties, respectively [24].

Our results revealed that individuals in the lowest tertile of DII were more likely to have a healthier lifestyle (more physical activity and less smoking), be female, and overweight, in spite of having lower energy intakes. The latter finding might be due to underreporting, which is more common in female and overweight/obese subjects [56], or reverse causality, wherein overweight/obese individuals tend to change their lifestyle and adhere to a healthy diet. Therefore, we further assessed the association between DII and psychosomatic complaints profiles stratified by BMI and found no statistically significant association between any of the psychosomatic complaint profiles and DII in overweight/obese subjects, whereas in subjects with normal weight, a greater DII was associated with increased risk of psychological and neuro-skeletal somatic complaints and tended to increase the risk of gastrointestinal somatic complaints (data not shown). However, in the present study, we are not able to draw any conclusions relating to causality, and longitudinal studies are needed.

This study has some limitations. First, the cross-sectional design did not allow us to investigate cause-effect relationships. For example, it is not clear whether psychosomatic complaints precede adherence to a pro-inflammatory diet, or whether the inflammatory potential of the diet causes psychosomatic complaints. On the other hand, it is possible that individuals who suffer from psychosomatic complaints tend to consume healthier foods with more anti-inflammatory properties to alleviate their symptoms. Second, in the present study, only gastrointestinal symptoms were evaluated using adequate clinical and technical approaches to provide a medical explanation, whilst other somatic symptoms are only indicative of somatization. Third, the DII has not been validated as a proxy measure of the inflammatory potential of diet in our study population; however, the consistency of our findings with others in the context of mental disorders [23] may confirm its validity. Fourth, the sex-specific association between DII and psychosomatic complaints profiles needs more research to determine the potential modifying effect of sex. It is not clear at this stage whether the different associations are real or attributable to methodological limitations. However, it is possible that some differences in lifestyle factors between men and women, such as smoking habits or physical activity, may explain the sex differences in DII-psychosomatic complaints. Although the confounding effects of such variables have been taken into account, the residual effects of such confounders or unknown or unmeasured confounders may influence the results. Fifth, the population of the study may have the possibility of selection because we selected participants from the working population, such as workers on the university campus and in hospitals and health centers, but not from the general community-dwelling population. Additionally, the subjects included in the study were 2828 of 10,087 subjects (approximately participation rate, 28%); therefore, despite including people with various socioeconomic status, our findings might not be generalizable to other populations. Finally, our study relied on the use of self-reported data.

The strengths of this study include the novelty of the topic and the use of validated questionnaires to evaluate dietary intakes and psychosomatic complaints. In addition, although the study was conducted among university employees, the large sample size of the study population with wide variation in demographic variables may make our results generalizable to other populations.

## Conclusions

In conclusion, this study provides evidence regarding the relation between a pro-inflammatory diet and psychosomatic complaints. Considering the adverse health outcomes associated with psychosomatic disorders, as well as the huge burden on individuals and society and the socio-economic impact, particularly due to their psychological dependency and medically unexplained nature, there is a need to expand our knowledge in this context and to develop new dietary interventions to reduce the incidence of psychiatric disorders and, consequently, their adverse health outcomes.

## Supplementary information

**Additional file 1: Table S1.** The correlations of dietary inflammatory index and its components with different psychosomatic complaints profiles. **Table S2.** Crude and multivariable-adjusted odds ratio and 95% confidence interval for various psychosomatic complaints profiles across tertiles of vitamin C. **Table S3.** Comparison of psychosomatic complaints profiles’ scores and Dietary inflammatory index between men and women^1^.

## Data Availability

Not applicable.

## Abbreviations

ANCOVA: Analysis of covariance
ANOVA: Analysis of variance
BMI: Body mass index
CI: Confidence interval
DFQ: Dish-based food-frequency questionnaire
DII: Dietary inflammatory index
GPAQ: General practice activity questionnaire
IL-1β: Interleukin-1β
IL-6: Interleukin-6
NF-κβ: Nuclear factor kappa beta
OR: Odds ratio
PHQ: Patient health questionnaire
SLE: Stressful life events
TNF-α: Tumor necrosis factor-α
