# Supplementary material for: The relationship between dietary inflammatory index and psychosomatic complaints profiles: results from SEPAHAN cross-sectional study
Source: Biopsychosoc Med. 2019 Nov 6;13:27. doi: 10.1186/s13030-019-0169-9 (PMC6836464; doi:10.1186/s13030-019-0169-9)
Supplement: Supplementary file 1 — Additional file 1: Table S1. The correlations of dietary inflammatory index and its components with different psychosomatic complaints profiles. Table S2. Crude and multivariable-adjusted odds ratio and 95% confidence interval for various psychosomatic complaints profiles across tertiles of vitamin C. Table S3. Comparison of psychosomatic complaints profiles’ scores and Dietary inflammatory index between men and women1. [file 13030_2019_169_MOESM1_ESM.docx]

Table S1: The correlations of dietary inflammatory index and its components with different psychosomatic complaints profiles.

|  | **Psychological somatic complaints profile** **(n= 2629)** | **Gastrointestinal somatic complaints profile** **(n=2234)** | **Neuro-skeletal somatic complaints profile** **(n=1578)** | **Pharyngeal-respiratory somatic complaints profile (n=2640)** |
| --- | --- | --- | --- | --- |
| Dietary inflammatory index | 0.057 | 0.034 | 0.047 | 0.051 |
| Energy | -0.017 | -0.030 | -0.001 | 0.001 |
| Carbohydrate | -0.015 | -0.032 | -0.004 | -0.005 |
| Protein | -0.030 | -0.033 | -0.013 | -0.008 |
| Total fat | -0.014 | -0.020 | 0.004 | 0.006 |
| Cholesterol | -0.029 | -0.028 | -0.010 | -0.004 |
| Monounsaturated fatty acids | -0.008 | -0.018 | 0.001 | 0.007 |
| Polyunsaturated fatty acids | -0.015 | -0.020 | 0.008 | 0.011 |
| Saturated fat | -0.015 | -0.015 | 0.006 | -0.001 |
| n-3 fatty acids | -0.033 | -0.024 | 0.001 | -0.010 |
| n-6 fatty acids | -0.013 | -0.020 | 0.008 | 0.013 |
| Trans fat | -0.009 | -0.007 | 0.011 | 0.029 |
| Fiber | -0.045 | -0.045 | -0.028 | -0.030 |
| Thiamin | -0.016 | -0.022 | -0.015 | 0.000 |
| Riboflavin | -0.036 | -0.036 | -0.014 | -0.024 |
| Niacin | -0.015 | -0.020 | -0.014 | 0.006 |
| Vitamin B6 | -0.043 | -0.050 | -0.027 | -0.017 |
| Folic acid | -0.018 | -0.030 | -0.013 | 0.002 |
| Vitamin B12 | -0.026 | -0.027 | 0.007 | -0.014 |
| Vitamin C | -0.063 | -0.058 | -0.050 | -0.075 |
| Vitamin A | -0.062 | -0.053 | -0.041 | -0.047 |
| Vitamin D | -0.029 | -0.025 | -0.005 | 0.002 |
| Vitamin E | -0.015 | -0.024 | -0.002 | 0.010 |
| β-carotene | -0.062 | -0.051 | -0.041 | -0.046 |
| Iron | -0.016 | -0.029 | -0.012 | 0.008 |
| Magnesium | -0.034 | -0.042 | -0.019 | -0.015 |
| Selenium | -0.021 | -0.023 | -0.015 | 0.002 |
| Zinc | -0.026 | -0.032 | -0.007 | -0.003 |
| Onions | -0.052 | -0.058 | -0.020 | -0.012 |
| Tea | 0.033 | 0.027 | 0.020 | 0.017 |
| Caffeine | 0.039 | 0.039 | 0.013 | 0.009 |

Table S2- Crude and multivariable-adjusted odds ratio and 95% confidence interval for various psychosomatic complaints profiles across tertiles of vitamin C.

|  | **Tertiles of vitamin C** | | | **P trend**^1^ |
| --- | --- | --- | --- | --- |
|  | **1** | **2** | **3** |  |
| n | 870 | 878 | 881 |  |
| **Psychological somatic complaints profile** |  |  |  |  |
| Crude | 1 (Reference) | 0.79 (0.65, 0.95) | 0.72 (0.60, 0.87) | 0.001 |
| Multivariate adjusted^2^ | 1 (Reference) | 0.78 (0.60, 1.01) | 0.76 (0.58, 1.002) | 0.056 |
| **Gastrointestinal somatic complaints profile** |  |  |  |  |
| Crude | 1 (Reference) | 0.77 (0.63, 0.95) | 0.67 (0.55, 0.82) | <0.0001 |
| Multivariate adjusted^2^ | 1 (Reference) | 0.82 (0.63, 1.06) | 0.77 (0.58, 1.02) | 0.068 |
| **Neuro-skeletal somatic complaints profile** |  |  |  |  |
| Crude | 1 (Reference) | 0.86 (0.67, 1.10) | 0.69 (0.54, 0.88) | 0.003 |
| Multivariate adjusted^2^ | 1 (Reference) | 0.95 (0.68, 1.32) | 0.76 (0.54, 1.07) | 0.109 |
| **Pharyngeal -respiratory somatic complaints profile** |  |  |  |  |
| Crude | 1 (Reference) | 0.80 (0.67, 0.97) | 0.63 (0.52, 0.76) | <0.0001 |
| Multivariate adjusted^2^ | 1 (Reference) | 0.78 (0.62, 0.99) | 0.63 (0.49, 0.82) | <0.0001 |

^1^ From Mantel-Haenszel extension chi-square test.

^2^Adjusted for age, energy, marital status, education, smoking, physical activity, BMI, anti-psychotropic medicines, medical history of diseases and stressful life events

Table S3 Comparison of psychosomatic complaints profiles’ scores and Dietary inflammatory index between men and women^1^.

|  | Psychological | Gastrointestinal | Neuro-skeletal | Pharyngeal-respiratory | Dietary inflammatory index |
| --- | --- | --- | --- | --- | --- |
| **Crude mean** |  |  |  |  |  |
| Men | 5.83±0.06 | 6.98±0.06 | 6.81±0.17 | 4.01±0.03 | -1.80±0.04 |
| Women | 6.70±0.06 | 7.63±0.06 | 8.49±0.06 | 4.12±0.02 | -2.09±0.03 |
| P^2^ | <0.0001 | <0.0001 | <0.0001 | 0.004 | <0.0001 |
| **Adjusted mean^3^** |  |  |  |  |  |
| Men | 5.38±0.30 | 6.22±0.26 | 6.21±0.26 | 3.56±0.13 | -1.72±0.05 |
| Women | 6.66±0.07 | 7.67±0.06 | 8.45±0.06 | 4.13±0.03 | -2.15±0.03 |
| P^4^ | <0.0001 | <0.0001 | <0.0001 | <0.0001 | <0.0001 |

^1^Values are mean±SE.

^2^Derived from independent samples t-test.

^3^Adjusted for age, energy, marital status, education, smoking, physical activity, BMI, anti-psychotropic medicines, medical history of diseases and stressful life events.

^4^Derived from analysis of covariance.
